# EVERREST prospective study: a 6-year prospective study to define the clinical and biological characteristics of pregnancies affected by severe early onset fetal growth restriction

**DOI:** 10.1186/s12884-017-1226-7

**Published:** 2017-01-23

**Authors:** Rebecca Spencer, Gareth Ambler, Jana Brodszki, Anke Diemert, Francesc Figueras, Eduard Gratacós, Stefan R. Hansson, Kurt Hecher, Angela Huertas-Ceballos, Neil Marlow, Karel Marsál, Eva Morsing, Donald Peebles, Carlo Rossi, Neil J. Sebire, John F. Timms, Anna L. David, EVERREST Consortium

**Affiliations:** 1 Institute for Women’s Health, University College London, London, UK; 2 Department of Statistical Science, University College London, London, UK; 3 Department of Clinical Sciences Lund, Obstetrics and Gynecology, Skane University Hospital, Lund University, Lund, Sweden; 4 Obstetrics and Fetal Medicine Unit, University Medical Centre Hamburg-Eppendorf, Hamburg, Germany; 5 BCNatal, Hospital Clinic and Hospital Sant Joan de Deu, University of Barcelona, CIBERER and IDIBAPS, Barcelona, Spain; 6 Neonatal Medicine, University College London Hospitals, London, UK; 7 Institute for Women’s Health, University College London and NIHR University College London Hospitals Biomedical Research Centre, London, UK; 8 Magnus Life Science, London, UK; 9 Paediatric and Developmental Pathology, Great Ormond Street Hospital, London, UK

**Keywords:** Fetal growth restriction, Prospective cohort, Ultrasound biometry, Doppler ultrasound, Angiogenic, Prediction, Outcome, Uteroplacental, Placental insufficiency

## Abstract

**Background:**

Fetal growth restriction (FGR) is a serious obstetric condition for which there is currently no treatment. The EVERREST Prospective Study has been designed to characterise the natural history of pregnancies affected by severe early onset FGR and establish a well phenotyped bio-bank. The findings will provide up-to-date information for clinicians and patients and inform the design and conduct of the EVERREST Clinical Trial: a phase I/IIa trial to assess the safety and efficacy of maternal vascular endothelial growth factor (VEGF) gene therapy in severe early onset FGR. Data and samples from the EVERREST Prospective Study will be used to identify ultrasound and/or biochemical markers of prognosis in pregnancies with an estimated fetal weight (EFW) <3rd centile between 20+0 and 26+6 weeks of gestation.

**Methods:**

This is a 6 year European multicentre prospective cohort study, recruiting women with a singleton pregnancy where the EFW is <3rd centile for gestational age and <600 g at 20+0 to 26+6 weeks of gestation. Detailed data are collected on: maternal history; antenatal, peripartum, and postnatal maternal complications; health economic impact; psychological impact; neonatal condition, progress and complications; and infant growth and neurodevelopment to 2 years of corrected age in surviving infants. Standardised longitudinal ultrasound measurements are performed, including: fetal biometry; uterine artery, umbilical artery, middle cerebral artery, and ductus venosus Doppler velocimetry; and uterine artery and umbilical vein volume blood flow. Samples of maternal blood and urine, amniotic fluid (if amniocentesis performed), placenta, umbilical cord blood, and placental bed (if caesarean delivery performed) are collected for bio-banking. An initial analysis of maternal blood samples at enrolment is planned to identify biochemical markers that are predictors for fetal or neonatal death.

**Discussion:**

The findings of the EVERREST Prospective Study will support the development of a novel therapy for severe early onset FGR by describing in detail the natural history of the disease and by identifying women whose pregnancies have the poorest outcomes, in whom a therapy might be most advantageous. The findings will also enable better counselling of couples with affected pregnancies, and provide a valuable resource for future research into the causes of FGR.

**Trial registration:**

NCT02097667 registered 31^st^ October 2013.

**Electronic supplementary material:**

The online version of this article (doi:10.1186/s12884-017-1226-7) contains supplementary material, which is available to authorized users.

## Background

Fetal growth restriction (FGR) is a serious condition affecting about 8% of all pregnancies and contributing to 30% of stillbirths [1]. As yet there is no therapy that improves fetal growth in utero, thus current management is to deliver the fetus before intrauterine death or irreversible organ damage occurs [2]. This is particularly challenging in early onset FGR, where delivery adds additional risks to the baby from extremely preterm birth, with its own attendant short and long-term complications [3–5]. Furthermore FGR may be detected when the estimated fetal weight (EFW) is below 500 g, a situation considered by many to be non-viable. FGR is most commonly due to three principal factors: a) maternal diseases such as infections; b) fetal chromosomal, genetic, or structural anomalies; and, most often, c) placental insufficiency. Placental insufficiency manifests as inadequate uteroplacental blood flow on ultrasound scan and maternal vascular malperfusion (MVM) on placental histology [6].

We are currently developing a treatment for FGR caused by placental insufficiency [orphan designation EU/3/14/1415], using maternal adenovirus gene therapy to increase expression of vascular endothelial growth factor (VEGF) protein in the uterine arteries. VEGF is secreted by the placenta, induces vasodilatation, and mediates vasculogenesis and angiogenesis [7, 8]. In FGR, however, maternal serum levels of VEGF are significantly lower than in normal pregnancy [9, 10]. Previous studies in normal sheep pregnancy show that administering adenovirus VEGF gene therapy (Ad.VEGF) into the maternal uterine arteries increases uterine artery blood volume flow long-term, causes nitric oxide release and relaxes the vessels [11–13]. Further studies in sheep and guinea pig models of FGR have shown that administering Ad.VEGF gene therapy into the maternal uterine arteries safely increases fetal growth [14, 15].

The EVERREST Consortium plans to carry out a phase I/IIa trial to assess the safety and efficacy of maternal uterine artery injection of Ad.VEGF in women with pregnancies affected by severe early onset FGR. This will be called the EVERREST Clinical Trial. For the first-in-woman trial of maternal gene therapy the eligibility criteria will be designed to identify severely affected pregnancies, where the balance of risk and potential benefit is most favourable. These pregnancies will naturally have high rates of maternal, fetal, and neonatal complications. The safety and efficacy of the intervention in the trial pregnancies will need to be compared with data from a cohort of similar severely affected pregnancies that do not undergo intervention.

Several prospective cohort studies have investigated the outcomes of pregnancies where the fetus was found to be small in mid-pregnancy [16–21]. Recent outcome data have also been provided by the Trial of Umbilical and Fetal Flow in Europe (TRUFFLE), a randomised controlled trial comparing different indications for delivery in pregnancies diagnosed with severe FGR from 26 weeks of gestation onwards [22, 23]. However, none of these studies includes the level of detailed maternal, fetal, and infant outcome data required to properly assess safety in the EVERREST Clinical Trial [24]. A detailed study of the natural history of pregnancies affected by severe early onset FGR is therefore required.

The EVERREST Prospective Study will clinically and biochemically characterise a cohort of pregnancies affected by severe early onset FGR across the four European centres that will be taking part in the EVERREST Clinical Trial. A database will be created containing information about antenatal investigations, maternal complications, fetal outcome and neonatal progress. This will be linked to a bio-bank containing samples including maternal blood and umbilical cord blood as well as tissue from the placenta, placental bed, and the myometrium. These samples will be used to investigate the natural history and biological mechanisms which underlie severe early onset FGR and, in conjunction with the database, will be used to investigate potential biomarkers for fetal and neonatal outcome.

## Methods

### Aims

1. To characterise the natural history of pregnancies affected by severe early onset FGR in order to:
  - Provide up-to-date knowledge for clinicians and future patients.
  - Interpret the safety of maternal growth factor gene therapy in a future clinical trial.
2. To establish a bio-bank of well phenotyped samples that can be used to investigate the biological mechanisms underlying severe early onset FGR.
3. To identify ultrasound and/or biochemical markers of prognosis in pregnancies with an EFW <3rd centile between 20+0 and 26+6 weeks of gestation.

### Design

This is a 6-year European multicentre prospective cohort study. The design and publication of this protocol has been performed in line with the ‘strengthening the reporting of observational studies in epidemiology’ (STROBE) guidelines [25, 26].

### Study population

#### Inclusion criteria

- Live singleton fetus.
- Estimated fetal weight <600 g and <3rd centile for gestational age as defined by local criteria.
- Gestational age at diagnosis 20+0 to 26+6 weeks of gestation, based on ultrasound and/or last menstrual period.
- Informed consent given by the participant and signed consent form completed.

#### Exclusion criteria

- Multiple pregnancy.
- Maternal age under 18 years.
- Known abnormal karyotype at enrolment.
- Known major fetal structural abnormality at enrolment defined as a lethal, incurable or curable severe abnormality with a high risk of residual handicap [27].
- Indication for immediate delivery.
- Women who lack the capacity to give informed consent.
- Any medical or psychiatric condition which compromises the woman’s ability to participate.
- Maternal HIV or hepatitis B or C infection.
- Premature preterm rupture of membranes before enrolment.

#### Study setting

Participants are recruited from University College Hospital London, University Medical Centre Hamburg-Eppendorf, Maternal-Fetal Unit Hospital Clinic Barcelona, and the Skane University Hospital, Lund. All four sites are tertiary referral centres for their surrounding area, performing 12,000 to 20,000 fetal ultrasound scans per year.

### Study procedures

**Fig. 1:**
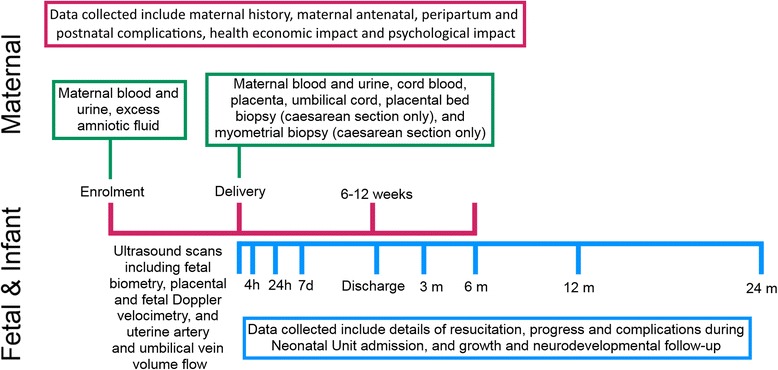
Participant timelines for the EVERREST Prospective Study

The study timeline is shown in Fig. 1.

#### Data collection

In order to fully characterise this cohort, an extensive data set is collected for each participant. This includes, but is not limited to, the majority of the optimal data set recommended by the Global Pregnancy CoLaboratory (COLAB) [28]. At enrolment maternal demographic, medical, obstetric, social, and family history are collected, as well as paternal demographic and family history and details of previously fathered pregnancies. Data are collected on the current pregnancy, including the results of ultrasound scans before and after enrolment, screening tests, invasive tests, and tests for maternal infection.

Data are collected about maternal antenatal, perinatal, and postnatal complications. Perinatal data are collected about the delivery, any maternal medication, the condition of the newborn and any need for resuscitation. For liveborn infants, data are collected at 4, 24 h, and 7 days of life as well as at discharge from the Neonatal Unit, if applicable. This includes data on cardiovascular, metabolic and respiratory status, growth, feeding, cranial ultrasound findings, retinopathy screening, and any medical or surgical complications. Surviving infants and children undergo neurodevelopmental follow-up until 2 years of age (corrected for prematurity, Table 1). Detailed health economic data are collected at approximately 6 weeks and 6 months post-delivery, including maternal and infant health service use and personal financial costs.

**Table 1:** EVERREST Prospective Study infant neurodevelopmental follow-up plan

Age (corrected for prematurity)	3 months	6 months	12 months	24 months
Growth and health	X	X	X	X
Prechtl video assessment	X			
Amiel Tison neurological assessment, hearing, vision, and general assessment of development	X	X		
Hammersmith neuroassessment			X	X
Bayley III			X	X
Gross Motor Function Classification System (GMFCS)				X
Manual Ability Classification System (MACS)				X

All data are entered onto a central MACRO database using the participant’s unique Participation Identification Number. This password protected database is stored on servers based at University College London, which are protected by thorough electronic and physical security measures. Within the database electronic Case Report Forms include internal data checks for validity and consistency, and regular data checks are performed by the EVERREST Study Manager.

#### Ultrasound measurements

The timing of ultrasound scans is determined clinically, with fetal biometry recorded on the database no more than once a week. All ultrasound measurements are performed according to a Standard Operating Procedure (SOP, see Additional file 1). Fetal biometry includes head and abdominal circumference, both measured using an ellipse, and femur length, all measured according to the guidelines of the International Society of Ultrasound in Obstetrics and Gynecology (ISUOG) [29]. Doppler ultrasound measurements are performed with the Doppler gate across the whole width of the vessel, the angle of insonation below 30°, with measurements taken from three or more consecutive uniform waveforms. Pulsatility index (PI) is recorded for the umbilical artery (from a free-floating portion), the middle cerebral artery, and the ductus venosus. End-diastolic flow in the umbilical artery and the a wave of the ductus venosus are characterised as positive, absent, or reversed. Uterine artery volume blood flow is measured after the participant has been resting for at least 20 min. Each uterine artery is visualised using power Doppler, using the minimum power setting needed to delineate the vessel walls. The vessel diameter is measured perpendicular to the vessel during systole, between the outer aspects of the lumen, and the waveform is recorded to measure time averaged mean (TAMEAN), PI, and to define the presence or absence of notching as described [30]. Umbilical vein volume blood flow is recorded from an intra-abdominal section of the umbilical vein, with the TAMEAN recorded with the angle of insonation as close as possible to 0° and the vessel diameter measured perpendicular to the lumen, between the inner aspects of the vessel walls [31].

Because neither uterine artery volume flow nor umbilical vein volume flow are routinely performed in any of the centres, a validation system has been established for these measurements. Within each site the local ultrasound lead reviews images taken by all members of staff performing EVERREST study measurements, both when they first scan a study patient and then annually thereafter. These images are scored according to predefined criteria, with further training and review if images are unsatisfactory. Once a year the local ultrasound lead must submit three sets of assessed images, along with the score they gave to each, to the study ultrasound lead for review.

#### Sample collection

Sample collection and processing procedures are performed according to the laboratory manual and SOPs. Maternal blood is collected at enrolment and delivery for plasma, citrated plasma, serum, RNA (PAXgene® tube), full blood count, renal and liver function, and lactate dehydrogenase. At enrolment maternal whole blood is stored for DNA. Maternal urine is collected at enrolment and delivery with aliquots of whole urine, urine centrifuged at slow and fast speeds, and the pellet from centrifugation. Any excess amniotic fluid from amniocentesis is also stored. At delivery umbilical cord blood is collected for arterial and venous gases, blood spot cards, plasma, and serum (assuming sufficient volume can be collected). Placental samples are collected from two areas of the placental parenchyma, each midway between the cord insertion and the margin in areas free from macroscopic infarcts. From each area full thickness samples are collected for histological analysis, and samples from the placental villous parenchyma (maternal and fetal surfaces removed) snap frozen and/or stored in RNALater®. For women undergoing delivery by caesarean section a placental bed biopsy is also collected and processed for histological analysis.

#### Participant experience

A recent amendment in the UK has extended the study to explore the impact on women of experiencing a pregnancy complicated by early onset FGR and of participating in research during pregnancy. At enrolment women are asked to complete the state section of the State-Trait Anxiety Inventory (STAI) [32]. This is repeated at 6–12 weeks postnatally, along with the Edinburgh Postnatal Depression Scale (EPDS) [33] and the 2008 version of the Perinatal Posttraumatic Stress Disorder Questionnaire (PPQ) [34]. In addition, all study participants who have consented to ongoing contact with the study have been invited to take part in a semi-structured qualitative interview sub-study and to complete a modified version of the Reactions to Research Participation Questionnaire (RRPQ) [35].

### Sample analyses

Three initial analysis strategies are planned for maternal blood samples taken at study enrolment:

1. Targeted angiogenic and anti-angiogenic markers: soluble FMS-like tyrosine kinase 1 (sFlt-1) and placental growth factor (PlGF) will be measured using the Roche Elecsys® automated electrochemiluminescence assay, VEGF-A, VEGF-D, soluble VEGF receptor-2, soluble endoglin, and neuropilin 1 will be measured using R&D Systems enzyme-linked immunosorbent assays (ELISAs).
2. Semi-targeted vascular markers: 92 serum analytes implicated in cardiovascular disease will be measured on the Olink ProSEEK platform, using DNA primer-tagged antibody pairs in a proximity extension assay to provide very sensitive (1 uL serum) and specific measurements.
3. Non-targeted proteomic analysis: a mass spectrometry-based serum profiling approach will be employed for the discovery of potential new biomarkers. Serum samples will be pooled into good and poor outcome groups and subjected to an established proteomic profiling workflow involving immunodepletion of the 12 most abundant serum proteins, tryptic digestion, 6-plex tandem mass tagging (TMT), and two-dimensional liquid chromatography (high and low pH reversed-phase) linked to quantitative tandem mass spectrometry. Candidate selection from the combined dataset will be based on standard statistical tests of fold-change, MS data quality and known function. Selected candidates will be further verified in the individual discovery samples using commercial ELISAs.

### Outcomes

The first aim of the study, characterising the natural history of severe early onset FGR, will involve multiple descriptive outcomes. The second aim, establishing a bio-bank, involves the creation of a resource for ongoing research with numerous potential endpoints. For the third aim, identifying prognostic markers, there will be two endpoints:

*Primary Endpoint*: Poor outcome, defined as fetal or neonatal death, including death in utero and within the first 28 days of life.

*Secondary Endpoint*: Time from enrolment to the end of pregnancy, defined as either diagnosis of stillbirth or delivery.

### Statistical analysis

Characterising the natural history of severe early onset FGR (Aim 1) will involve descriptive statistical methods, including means for numerical variables and proportions for binary or categorical variables. An initial analysis is planned for the first 65 study participants to help refine the inclusion criteria for the EVERREST Clinical Trial.

The association between individual factors and outcome will be analysed using t tests and chi-squared tests, and non-parametric tests used if required (Mann–Whitney U tests and Fisher’s exact tests). Logistic regression modelling will be used to investigate whether individual and combinations of factors can be used to estimate poor outcome (primary endpoint). These factors include ultrasound findings, levels and ratios of angiogenic factors, and potential markers identified through the semi-targeted and proteomic approaches. Due to the relatively small sample size, a forward selection algorithm will be employed, and it is likely that each model will only sustain a maximum of two or three independent variables. Models will be tested for goodness of fit and discrimination using leave-one out cross validation and bootstrapping.

Survival analysis, including Cox proportional hazards regression, will be used to investigate the predictive value of individual and combined factors for the secondary outcome measure of time until the end of pregnancy.

An a priori sample size calculation is not provided, as there is currently no reliable data on expected means and standard deviations of candidate markers within such a cohort, and no data on whether and how these would differ by outcome. Within our current sample of 65, with 5 withdrawals and 18 poor outcomes, there is a ratio of good to poor outcomes of 2.33. This sample will give an 80% power to detect a standardised effect size of 1.0 to a significance level of 0.05 [36]. The potential for type 1 error, given the multiple comparisons, or type 2 error, given the small sample size, will be borne in mind when interpreting the study results.

## Discussion

Developing novel maternal and fetal therapies raises many challenges [37]. By characterising the natural history of severe early onset FGR the EVERREST Prospective study will make it possible to assess the safety and efficacy of maternal Ad.VEGF gene therapy in an early phase clinical trial. The identification of prognostic markers may also help refine the eligibility criteria for later phase clinical trials, improving the chance of successful translation into the clinic. The natural history data will also be available for comparison with other novel therapies that may be in development [2].

Establishing this study within the context of a pan-European collaboration has provided both challenges and benefits. Development of the study protocol and SOPs has highlighted the variation in practice that exists between clinical fetal medicine units. Routine clinical practice in the four study sites uses different methods of measuring fetal biometry, different formulae for EFW, and different reference ranges for EFW and Doppler measurements. These have been harmonised for the EVERREST Prospective Study, providing us with the basis to move forwards for the EVERREST Clinical Trial. Through establishing the ultrasound validation system we have also addressed the potential for variation in practice in a multicentre study.

Defining the eligibility criteria was complicated by the considerable heterogeneity in the way the term FGR is used and defined. An EFW below the third centile has been chosen as the inclusion criteria for the EVERREST Prospective Study in order to identify the smallest fetuses, at the highest risk of adverse perinatal outcome [17]. This is consistent with the recent Delphi consensus on the definition of early placental FGR [38]. An upper limit of 600 g and 26+6 weeks of gestation was included because Consortium members considered that above these parameters, delivery would be a more appropriate option than participation in the EVERREST Clinical Trial. The inclusion criteria do not include any Doppler ultrasound measurements, meaning that there is the potential for inclusion of pregnancies where the fetus is constitutionally small. This was a deliberate decision in order to avoid initially excluding women who subsequently manifested signs of placental insufficiency. More restrictive eligibility criteria are planned for the EVERREST Clinical Trial, and outcomes will be compared with women from prospective study who would have met these more stringent criteria.

An important feature of the EVERREST Prospective Study is the detailed maternal, fetal, and infant data that are being collected. The outcomes to be recorded were initially developed by pooling variables collected in other obstetric and neonatal studies across the four centres. Shortly after the launch of the study COLAB published their minimum and optimum data sets for studies on pre-eclampsia [28]. Many of these variables are applicable to all studies in pregnancy, and given the overlap between pre-eclampsia and FGR they are particularly relevant to the EVERREST Prospective Study. We therefore expanded the data collection to include the majority of their optimal data set. COLAB will shortly be launching a database incorporating their recommended data sets, which will be a valuable resource for future pregnancy studies.

The EVERREST Prospective Study has been designed to support the development and conduct of the EVERREST Clinical Trial, but we believe that its findings will have wider applications. By providing outcome data and prognostic markers for a severe early onset subset of FGR, this study is likely to improve future patient counselling and guide clinician’s management. The bio-bank of comprehensively phenotyped samples will also support future research into the biological mechanisms underlying FGR, and hopefully identify novel targets for future therapies.

## Additional file

Additional file 1: EVERREST Standard Operating Procedure for performing ultrasound examinations. (PDF 1019 kb)

## Abbreviations

Ad.VEGF: Adenoviral vector vascular endothelial growth factor gene therapy
COLAB: Global Pregnancy CoLaboratory
DNA: Deoxyribonucleic acid
EFW: Estimated fetal weight
ELISA: Enzyme-linked immunosorbent assay
EPDS: Edinburgh postnatal depression scale
EVERREST: Does vascular endothelial growth factor gene therapy safely improve outcome in severe early-onset fetal growth restriction?
FGR: Fetal growth restriction
GMFCS: Gross motor function classification system
HIV: Human immunodeficiency virus
ISUOG: International society of ultrasound in obstetrics and gynecology
MACS: Manual ability classification system
MVM: Maternal vascular malperfusion
PI: Pulsatility index
PlGF: Placental growth factor
PPQ: Perinatal posttraumatic stress disorder questionnaire
RNA: Ribonucleic acid
ROC: Receiver operating characteristic
RRPQ: Reactions to research participation questionnaire
sFlt-1: Soluble FMS-like tyrosine kinase 1
SOP: Standard operating procedure
STAI: State-trait anxiety inventory
TAMEAN: Time averaged mean
TMT: Tandem mass tagging
VEGF: Vascular endothelial growth factor

## References

[1] Lawn JE, Blencowe H, Pattinson R, Cousens S, Kumar R, Ibiebele I (2011). Stillbirths: Where? When? Why? How to make the data count?. Lancet.

[2] Spencer RN, Carr DJ, David AL (2014). Treatment of poor placentation and the prevention of associated adverse outcomes - what does the future hold?. Prenat Diagn.

[3] Marlow N, Wolke D, Bracewell MA, Samara M (2005). Neurologic and developmental disability at six years of age after extremely preterm birth. N Engl J Med.

[4] Brodszki J, Morsing E, Malcus P, Thuring A, Ley D, Marsal K (2009). Early intervention in management of very preterm growth-restricted fetuses: 2-year outcome of infants delivered on fetal indication before 30 gestational weeks. Ultrasound Obstet Gynecol.

[5] Morsing E, Asard M, Ley D, Stjernqvist K, Marsal K (2011). Cognitive function after intrauterine growth restriction and very preterm birth. Pediatrics.

[6] Khong TY, Mooney EE, Ariel I, Balmus NC, Boyd TK, Brundler MA, et al. Sampling and definitions of placental lesions: Amsterdam Placental Workshop Group Consensus Statement. Arch Pathol Lab Med. 2016;140(7):698-713. 10.5858/arpa.2015-0225-CC 27223167

[7] Brownbill P, Mills TA, Soydemir DF, Sibley CP (2008). Vasoactivity to and endogenous release of vascular endothelial growth factor in the in vitro perfused human placental lobule from pregnancies complicated by preeclampsia. Placenta.

[8] Maynard SE, Min JY, Merchan J, Lim KH, Li J, Mondal S (2003). Excess placental soluble fms-like tyrosine kinase 1 (sFlt1) may contribute to endothelial dysfunction, hypertension, and proteinuria in preeclampsia. J Clin Invest.

[9] Savvidou MD, Yu CK, Harland LC, Hingorani AD, Nicolaides KH (2006). Maternal serum concentration of soluble fms-like tyrosine kinase 1 and vascular endothelial growth factor in women with abnormal uterine artery Doppler and in those with fetal growth restriction. Am J Obstet Gynecol.

[10] Bersinger NA, Odegard RA (2005). Serum levels of macrophage colony stimulating, vascular endothelial, and placenta growth factor in relation to later clinical onset of pre-eclampsia and a small-for-gestational age birth. Am J Reprod Immunol.

[11] David AL, Torondel B, Zachary I, Wigley V, Abi-Nader K, Mehta V (2008). Local delivery of VEGF adenovirus to the uterine artery increases vasorelaxation and uterine blood flow in the pregnant sheep. Gene Ther.

[12] Mehta V, Abi-Nader KN, Peebles DM, Benjamin E, Wigley V, Torondel B (2012). Long-term increase in uterine blood flow is achieved by local overexpression of VEGF-A(165) in the uterine arteries of pregnant sheep. Gene Ther.

[13] Mehta V, Abi-Nader KN, Shangaris P, Shaw SW, Filippi E, Benjamin E (2014). Local over-expression of VEGF-DΔNΔC in the uterine arteries of pregnant sheep results in long-term changes in uterine artery contractility and angiogenesis. PLoS One.

[14] Carr D, Wallace JM, Aitken RP, Milne JS, Mehta V, Martin JF (2014). Uteroplacental adenovirus VEGF gene therapy increases fetal growth velocity in growth-restricted sheep pregnancies. Hum Gene Ther.

[15] Swanson A, Rossi C, Ofir K, Mehta V, Boyd M, Barker H, et al. Maternal therapy with Ad.VEGF-A165 increases fetal weight at term in a guinea pig model of fetal growth restriction. Hum Gene Ther. 2016;27(12):997–1007. 10.1089/hum.2016.046 27530140

[16] Hecher K, Bilardo CM, Stigter RH, Ville Y, Hackeloer BJ, Kok HJ (2001). Monitoring of fetuses with intrauterine growth restriction: a longitudinal study. Ultrasound Obstet Gynecol.

[17] Unterscheider J, Daly S, Geary MP, Kennelly MM, McAuliffe FM, O’Donoghue K, et al. Optimizing the definition of intrauterine growth restriction: the multicenter prospective PORTO Study. Am J Obstet Gynecol. 2013;208(4):290 e1-6. 10.1016/j.ajog.2013.02.007 23531326

[18] Bilardo CM, Wolf H, Stigter RH, Ville Y, Baez E, Visser GH (2004). Relationship between monitoring parameters and perinatal outcome in severe, early intrauterine growth restriction. Ultrasound Obstet Gynecol.

[19] Baschat AA, Cosmi E, Bilardo CM, Wolf H, Berg C, Rigano S (2007). Predictors of neonatal outcome in early-onset placental dysfunction. Obstet Gynecol.

[20] Odibo AO, Goetzinger KR, Cahill AG, Odibo L, Macones GA. Combined sonographic testing index and prediction of adverse outcome in preterm fetal growth restriction. Am J Perinatol. 2014;31(2):139–44. 10.1055/s-0033-1341574 PMC3932308 23546845

[21] Lawin-O’Brien AR, Dall’Asta A, Knight C, Sankaran S, Scala C, Khalil A, et al. Short term outcome of Periviable SGA: Is our counseling up to date? Ultrasound Obstet Gynecol. 2016;48(5):636–641. 10.1002/uog.15973 27854384

[22] Lees C, Marlow N, Arabin B, Bilardo CM, Brezinka C, Derks JB (2013). Perinatal morbidity and mortality in early-onset fetal growth restriction: cohort outcomes of the trial of randomized umbilical and fetal flow in Europe (TRUFFLE). Ultrasound Obstet Gynecol.

[23] Lees CC, Marlow N, van Wassenaer-Leemhuis A, Arabin B, Bilardo CM, Brezinka C, et al. 2 year neurodevelopmental and intermediate perinatal outcomes in infants with very preterm fetal growth restriction (TRUFFLE): a randomised trial. Lancet. 2015;385(9983):2162–72. 10.1016/S0140-6736(14)62049-3 25747582

[24] Spencer R, Carr D, David A (2015). Short-term outcome after antenatal diagnosis of severe early-onset fetal growth restriction. BJOG.

[25] Vandenbroucke JP, von Elm E, Altman DG, Gotzsche PC, Mulrow CD, Pocock SJ (2007). Strengthening the reporting of observational studies in epidemiology (STROBE): explanation and elaboration. PLoS Med.

[26] The PLOS Medicine Editors (2014). Observational studies: getting clear about transparancy. PLoS Med.

[27] Carvalho MHB, Brizot ML, Lopes LM, Chiba CH, Miyadahira S, Zugaib M (2002). Detection of fetal structural abnormalities at the 11–14 week ultrasound scan. Prenat Diagn.

[28] Myatt L, Redman CW, Staff AC, Hansson S, Wilson ML, Laivuori H (2014). Strategy for standardization of preeclampsia research study design. Hypertension.

[29] Salomon LJ, Alfirevic Z, Berghella V, Bilardo C, Hernandez-Andrade E, Johnsen SL (2011). Practice guidelines for performance of the routine mid-trimester fetal ultrasound scan. Ultrasound Obstet Gynecol.

[30] McKelvey A, Pateman K, Balchin I, Peebles DM, Rodeck CH, David AL. Total uterine artery blood volume flow rate in nulliparous women (TVFR) is associated with birthweight and gestation at delivery. Ultrasound Obstet Gynecol. 2017;49(1):54–60. 10.1002/uog.15917 26990029

[31] Acharya G, Wilsgaard T, Rosvold Berntsen GK, Maltau JM, Kiserud T (2005). Reference ranges for umbilical vein blood flow in the second half of pregnancy based on longitudinal data. Prenat Diagn.

[32] Meades R, Ayers S (2011). Anxiety measures validated in perinatal populations: a systematic review. J Affect Disord.

[33] Costeloe K, Hennessy E, Gibson AT, Marlow N, Wilkinson AR (2000). The EPICure study: outcomes to discharge from hospital for infants born at the threshold of viability. Pediatrics.

[34] Callahan JL, Borja SE (2008). Psychological outcomes and measurement of maternal posttraumatic stress disorder during the perinatal period. J Perinat Neonatal Nurs.

[35] Newman E, Willard T, Sinclair R, Kaloupek D (2001). Empirically supported ethical research practice: the costs and benefits of research from the participants’ view. Accountability in Research.

[36] Cohen J. Statistical Power analysis for behavioural sciences. 2nd ed. Hillsdale: Lawrence Erlbaum Associates; 1988.

[37] David A, Thornton S, Sutcliffe A, Williams P (2015). Scientific impact paper No. 50. Developing new pharmaceutical treatments for obstetric conditions.

[38] Gordijn SJ, Beune IM, Thilaganathan B, Papageorghiou A, Baschat AA, Baker PN, et al. Consensus definition for placental fetal growth restriction: a Delphi procedure. Ultrasound Obstet Gynecol. 2016;48(3):333–9. 10.1002/uog.15884 26909664

