# Supplementary material for: EVERREST prospective study: a 6-year prospective study to define the clinical and biological characteristics of pregnancies affected by severe early onset fetal growth restriction
Source: BMC Pregnancy Childbirth. 2017 Jan 23;17:43. doi: 10.1186/s12884-017-1226-7 (PMC5259830; doi:10.1186/s12884-017-1226-7)
Supplement: Additional file 1: — EVERREST Standard Operating Procedure for performing ultrasound examinations. (PDF 1019 kb) [file 12884_2017_1226_MOESM1_ESM.pdf]

UCL Clinical Trials Unit

Document Title:	**Standard Operating Procedure for performing ultrasound examinations**
Document Type:	SOP
Document Number:	EVE002
Version Number:	Version 3.0
Effective Date (training can commence):	23 March 2015
Date of Final Release:	20 March 2015
Review Date:	1 year from initial release and 2 years thereafter
(If applicable) Version of SOP being superseded:	2.0

Author	Title	Signature	Date
Dr. Anna David	Principal Investigator, Reader and Consultant in Obstetrics and Maternal Fetal Medicine	[signature]	17 March 2015.
**Reviewed by**		**Signature**	**Date**
Professor Donald Peebles	UCLH Co-Investigator, Professor and Head of the Research Department of Maternal Fetal Medicine		
Professor Karel Marsal	ULUND Principal Investigator, Professor at the Department of Obstetrics and Gynaecology		
Professor Kurt Hecher	UKE Principal Investigator, Professor and Chairman of the Department of Obstetrics and Fetal Medicine		
Dr. Eduard	IDIBAPS Principal Investigator,		

Gratacós	Head and Professor of the Department and Research Centre in Maternal Fetal Medicine		
Dr Francesc Figueras	Principal investigator for IDIBAPS Department and Research Centre in Maternal Fetal Medicine		
Anna Morka	Trial Manager	[illegible]	10 Mar 2015
Julie Bakobaki	Clinical Operations Manager	[illegible]	10 MARCH 2015
**Approvals**	**Title**	**Signature**	**Date**
Dr. Anna David	Chief Investigator	[illegible]	17 March 2015.

INTRODUCTION

Study Title: EVERREST- Developing a therapy for Fetal Growth Restriction.
A 6 year prospective study to define the clinical and biological characteristics of pregnancies affected by severe early onset Fetal Growth Restriction

Study ID: CTU/2013/060

PURPOSE

The purpose of this SOP is to ensure that ultrasound measurements recorded as part of the EVERREST Prospective Study are taken in a consistent manner and measurement errors are minimised.

RESPONSIBILITY

This procedure is applicable to any person carrying out an obstetric ultrasound examination on a participant in the EVERREST Prospective Study, where the findings of that ultrasound will be recorded as part of the study. This includes obstetric ultrasound examinations which form part of routine clinical care.

DOCUMENTATION

Ensure that all measurements taken for the purpose of the study are documented in clinical / electronic records to allow source verification, even if they do not form part of your routine protocols. These records should also include:

- Operator name
- Type of ultrasound machine and transducer used

DEFINITIONS

TAMean velocity: Time Averaged Mean velocity

TAMax velocity: Time Averaged Maximum velocity

PROCEDURES

1. Pulsed Wave Doppler Ultrasound: General advice

- Calculate pulsatility indices (PIs) from three or more consecutive waveforms obtained during minimal fetal activity and the absence of fetal breathing.
- Ask the mother to suspend her respiration just before recording the blood flow. This has been shown to reduce variability in the measurement.
- Use colour or colour power angiography to identify the vessels when measuring TAMean velocity and TAMax velocity.
- For measurement of TAMean and TAMax velocity place the Doppler gate to ensure the whole width of the vessel is included and keep the angle of insonation below 30°.
- Use a low wall filter (high-pass filter) available for all Doppler measurements. Record the cut-off level of the filter.
- Ensure that the ultrasound machine software is correctly identifying the lowest velocity during diastole. Where reversed end diastolic flow is present the lowest velocity should have a negative value.
- When selecting traced waveforms for analysis, ensure both markers are placed at the beginning of waveforms, to give correct time averaged measurements.
- Use colour power angiography to outline the vessel lumen for measurement of uterine artery diameter
- Use grey scale to identify vessel walls for measurement of umbilical vein diameter.

1.1. Umbilical Artery

- Measure umbilical artery PI from a free-floating portion of the umbilical cord.
- Use the waveforms recorded for this measurement to characterise the end-diastolic flow (EDF) as positive, absent or reversed.

1.2. Middle Cerebral Artery (MCA)

- In a transverse view of the fetal skull, at the level of the circle of Willis, visualise the MCA which is closest to the transducer / uppermost.
- Keep the angle of insonation as close to 0° as possible.
- Measure MCA PI from the proximal third of the MCA, close to its origin in the internal carotid artery.

1.3. Ductus Venosus (DV)

- Identify the DV in a mid-sagittal section of the fetal trunk or transverse section of the fetal abdomen, just after it branches from the umbilical vein.
- Measure the DV PI.
- Use the waveforms recorded for this measurement to characterise a wave as positive, absent or reversed.

1.4. Umbilical Vein (UV)

Measuring umbilical vein time average maximum (TAMax) Velocity

- Using colour or colour power angiography identify an intra-abdominal section of the umbilical vein, midway between the insertion of the cord into the abdominal cavity and the origin of the ductus venosus.
- Keep the angle of insonation as close to 0° as possible, and not more than 30°.
- Record the time average maximum (TAMax) velocity over 10 seconds while the mother is suspending her breathing.
- Take two further readings over the course of a minute..
- Use the Doppler spectrum recorded for this measurement to decide whether or not there are umbilical venous pulsations, defined as a diastolic decrease in blood velocity exceeding 15% of the baseline maximum. Record whether these pulsations are single or double (triphasic).

Measuring umbilical vein vessel diameter

- Obtain a view of the abdomen with umbilical vein in perpendicular to the USS beam ie horizontal
- Use grey scale to delineate a straight portion of the vessel between the ductus venosus and the abdominal wall
- Zoom in to enlarge the image so that the abdomen fills the screen
- Using grey scale measure the vessel diameter perpendicular to the lumen by placing the calipers "inner to inner", ie on the inside wall of the lumen.
- Take two further measurements

1.5. Uterine Arteries

Measuring uterine artery time average mean velocity (TAMean)

- Perform uterine artery Dopplers after the woman has been resting for at least 20 minutes after arrival in the department. This may include the time for the rest of the ultrasound scan.
- Identify each uterine artery in turn trans-abdominally by placing the probe on the lower quadrant of the abdomen, medial to the anterior superior iliac spine and angling medially.

- Use colour or colour flow angiography to visualise the uterine artery just proximal to the crossover with the external iliac artery.
- Keep the angle of insonation as close to 0° as possible, and not more than 30°.
- Widen the sample volume gate to include the whole width of the vessel so that the total blood velocity within the vessel is captured.
- Measure uterine artery PI and TAMean over a minimum of 3 and a maximum of 6 cardiac cycles. Ask the mother to suspend her breathing while the velocity is being recorded.
- Take two further readings over approximately a minute..
- Use the waveforms recorded for these measurements to decide whether or not there is notching. A notch is defined as a persistent decrease in blood flow velocity in early diastole, below the highest velocity during late diastole.

Measuring uterine artery vessel diameter

- Use colour power angiography to delineate outer aspects of the lumen of the vessel at low output power setting
- Keep the angle of insonation < 30°
- Measure the vessel diameter during systole
- The diameter should be measured perpendicular to the vessel walls at the outer aspects of the lumen, delineated by the colour power angiography pixels.
- Repeat twice more.

2. Fetal Biometry

2.1. Head Circumference (HC)

- Measure HC from a cross-sectional view of the fetal skull at the level of the thalamus, demonstrating the falx cerebri and cavum septi pellucidi but without any cerebellum visible.
- Measure HC directly using an ellipse fitted to the outer border of the skull.

2.2. Abdominal Circumference (AC)

- Measure AC from a transverse section of the fetal abdomen, demonstrating the umbilical vein at the level of the portal sinus and the fetal stomach. Neither the kidneys nor the heart should be visible and the abdomen should be as circular as possible.
- Measure AC directly using an ellipse fitted to the outer border of the abdomen.

2.3. Femur Length (FL)

- Measure FL with an angle of insonation of 90° to the longest axis and both ends of the ossified metaphysis visible. Measure from the ends of the ossified diaphysis. Take 3 measurements and record the longest.

3. Deepest Vertical Pool

- Keeping the probe perpendicular to the uterus, identify the deepest cord-free pocket of amniotic fluid. If required use colour flow mapping to ensure the pocket is cord-free.
- Measure the greatest vertical distance within this pocket that has nothing but amniotic fluid along the line of measurement.

4. Placental thickness

- Keeping the probe perpendicular to the uterus and the plane of the placenta, measure the placental thickness at the site of umbilical cord insertion, near the mid-placental portion. Take 3 measurements and record the mean.
- If the placental cord insertion is lateral or marginal, then measure the placental thickness at its widest part.

REFERENCES

Berkley E, Chauhan SP, Abuhamad A. Doppler assessment of the fetus with intrauterine growth restriction. American journal of obstetrics and gynecology. 2012;206(4):300-8.

Bhide A, Acharya G, Bilardo CM, Brezinka C, Cafici D, Hernandez-Andrade E, et al. ISUOG practice guidelines: use of Doppler ultrasonography in obstetrics. Ultrasound in obstetrics & gynecology : the official journal of the International Society of Ultrasound in Obstetrics and Gynecology. 2013;41(2):233-39.

Cheema R, Bayoumi Z, Gudmundsson S. Multivascular Doppler surveillance in high risk pregnancies. The Journal of Maternal-Fetal and Neonatal Medicine. 2012;25(7):970-4.

Chitty LS, Altman DG, Henderson A, Campbell S. Charts of fetal size: 3. Abdominal measurements. British journal of obstetrics and gynaecology. 1994;101(2):125-31.

Figueras F, Benavides A, Del Rio M, Crispi F, Eixarch E, Martinez JM, et al. Monitoring of fetuses with intrauterine growth restriction: longitudinal changes in ductus venosus and aortic isthmus flow. Ultrasound in obstetrics & gynecology : the official journal of the International Society of Ultrasound in Obstetrics and Gynecology. 2009;33(1):39-43.

Gómez O, Figueras F, Fernández S, Bennasar M, Martínez JM, Puerto B, et al. Reference ranges for uterine artery mean pulsatility index at 11–41 weeks of gestation. Ultrasound in Obstetrics and Gynecology. 2008;32(2):128-32.

Hecher K, Campbell S, Snijders R, Nicolaides K. Reference ranges for fetal venous and atrioventricular blood flow parameters. Ultrasound in Obstetrics and Gynecology. 1994;4(5):381-90.

Hernandez-Andrade E, Crispi F, Benavides-Serralde JA, Plasencia W, Diesel HF, Eixarch E, et al. Contribution of the myocardial performance index and aortic isthmus blood flow index to predicting mortality in preterm growth-restricted fetuses. Ultrasound in obstetrics & gynecology : the official journal of the International Society of Ultrasound in Obstetrics and Gynecology. 2009;34(4):430-6.

Marsal K, Persson PH, Larsen T, Lilja H, Selbing A, Sultan B. Intrauterine growth curves based on ultrasonically estimated foetal weights. Acta paediatrica (Oslo, Norway : 1992). 1996;85(7):843-8.

Rigano S, Bozzo M, Ferrazzi E, Bellotti M, Battaglia FC, Galan HL. Early and persistent reduction in umbilical vein blood flow in the growth-restricted fetus: a longitudinal study. Am J Obstet Gynecol 2001;185:834-8

Salomon LJ, Alfirevic Z, Berghella V, Bilardo C, Hernandez-Andrade E, Johnsen SL, et al. Practice guidelines for performance of the routine mid-trimester fetal ultrasound scan. Ultrasound in obstetrics & gynecology : the official journal of the International Society of Ultrasound in Obstetrics and Gynecology. 2011;37(1):116-26.

HISTORY OF REVISIONS

SOP Name	Version No.	Effective Date	Reason for change	Author(s)
EVE002 Standard Operating Procedure for performing ultrasound examinations	1.0	15th Sept 2014	• Umbilical vein inappropriately included PI and did not state to take duplicate readings of TAMEAN<br>• Francesc Figueras added as co-investigator	Dr. Rebecca Spencer/EVERREST Collaborators
	2.0		Unclear description of measurement of TAMean and diameter of uterine artery and peak velocity and diameter of umbilical vein. Clarified to bring into line with validation protocol for these measurements	Dr. Anna David

Appendix 1

Ultrasound Validation Scheme and Scoring System

Definitions:

TAMean velocity: Time Averaged Mean velocity

TAMax velocity: Time Averaged Maximum velocity

The EVERREST study is assessing fetal and maternal Dopplers as part of the prospective study and the intervention trial. Since the primary and secondary outcome measures of the trial are dependent on these Doppler measurements it is important that assessment is uniform across the study sites. Some of the proposed Doppler examinations in EVERREST are not routinely performed: Uterine artery volume blood flow (TAMean velocity, TAMax velocity and diameter), umbilical vein volume blood flow (TAMean velocity, TAMax velocity and diameter). A quality assessment and validation system has therefore been developed to ensure uniformity and correct technique across the sites.

A detailed protocol and description of all the Doppler measurements to be taken has been agreed by the participating clinical centres . Fetal biometry measurements and measurement of ductus venosus, middle cerebral artery and umbilical artery Doppler indices are familiar to fetal medicine specialists and do not form part of this validation scheme.

All participating sites and ultrasonographers/fetal medicine specialists will be provided with a presentation including video ultrasound clips describing and illustrating the measurement techniques. Each measurement has a set of defining components that have to be achieved in order to be considered "technically appropriate". A lead fetal medicine specialist (Local Ultrasound Lead) will be appointed at each center and a supervising specialist (Study Ultrasound Lead) will be appointed at the main study center (UCLH) to coordinate the validation process.

Table: Local and Study Ultrasound Leads

Centre	Local Ultrasound Lead
UCLH	Rebecca Spencer
IDIBAPS	Francesc Figueras
UKE	Anke Diemert
Lund	Jana Brodszki

Images to be submitted including measurement taken:

Uterine artery TAMean velocity

Uterine artery diameter

Umbilical vein TAMax velocity

Umbilical vein diameter

After performing their first scan for the EVERREST study each fetal medicine specialist/ultrasonographer should submit one example image of each of the uterine artery and umbilical vein Doppler measurements that they have taken as part of the study, to the Local Ultrasound Lead for review. The Local Ultrasound Lead should ascertain that the images taken and measurements done are as per the EVERREST ultrasound protocol. A score will be given according to the specific defining components of each measurement, as described below. In the case of poor technique or deviation from EVERREST guidelines, the Local Ultrasound Lead should notify the person being assessed and guide them on proper technique. Then, a second set of images, taken after the initial assessment and proper technique guidance, should be submitted to the Local Ultrasound Lead for sign off.

Subsequently once a year at each center, each of the fetal medicine specialists/ultrasonographers that are signed off to scan for the EVERREST study should submit one example image as above to the Local Ultrasound Lead.

All recordings and images of exams sent electronically off site to the CCTU **Must** be encrypted to **AES-256** details. Details of the encryption process are listed below under the section: Guidance on encryption for exams.

Once a year each center should be assessed by the Study Ultrasound Lead. The Local Ultrasound Lead should submit the last three exams that they had assessed, including the score given, for evaluation.

Scoring System

The scoring system is simple. A point is given for every defining component that is present in the measurement. The final score is a fraction made up of the number of defining components achieved out of the total defining components of each specific measurement. For a practitioner to be signed off to start they should achieve a score of 100% (ie 5 out of 5 correct).

SCORING SYSTEM

Umbilical Vein Doppler Velocity

Measurements –3 x TAMax velocity

**Defining Component**	**Score**
Colour or colour power angiography to visualize umbilical vein midway between abdominal wall and ductus venosus origin	1
Angle of insonation < 30°, as close to 0°	1
Sample volume gate widened to include the whole width of the vessel	1
Record velocity over 10 seconds	1
Define if umbilical venous pulsations (diastolic decrease in blood velocity exceeding 15% of the baseline maximum)	1

Umbilical Vein Diameter

Measurements – 3 x vessel diameter

**Defining Component**	**Score**
Visualise a straight portion of the vessel in grey scale midway between the abdominal wall and ductus venosus	1
Umbilical vein is perpendicular to USS beam ie horizontal	1
Maximum magnification (abdomen fills the view)	1
Measurement is taken perpendicular to lumen with UV horizontal	1
Calipers placed at the inner edges of the vessel wall (on to on)	1

Uterine Artery Doppler Velocity

Description of plane - Probe placed longitudinally just medial to anterior superior iliac spine in the lower lateral quadrant of abdomen, angled medially. Color flow mapping used to identify the uterine artery as it is seen crossing the external iliac artery.

Measurements – 3 x TAMean

Defining Component	Score
Colour or colour power angiography used to identify the uterine artery	1
Sample volume placed proximally downstream from<br><br>crossover of uterine artery and external iliac artery	1
Angle of insonation < 30°	1
Sample volume gate wide enough to include the whole width of the vessel	1
A minimum of 3 and a maximum of 6 cardiac cycles selected	1

Uterine Artery Diameter

Measurements – 3 x vessel diameter

Defining Component	Score
Use colour power angiography to delineate the walls of vessel at low output power setting	1
Angle of insonation < 30°	1
Calipers to measure the diameter are placed at the outer aspects of the vessel lumen delineated by the colour power angiography pixels	1
Measurement taken perpendicular to lumen	1
Measure during systole	1

Guidance on encryption for Examination Images

All ultrasound exams sent outside the original organisation needs to encrypted to ensure that only restricted personnel have access to this information. This can be done by using the encryption software 'Zip-7'; *this free software that is available online* (http://www.7-zip.org/download.html) and can be downloaded as either the *.exe* or *.msi*

If you have any issues downloading the software please contact your local IT support.

Please ensure you have been provided with a password by CCTU. Please contact the Trial Manager if you have not received a password. The password should ideally be of 8 characters and a mixture of mixed case letters, numbers and punctuation. Please only use the Password provided by the EVERREST Trial Manager.

Once the software has been downloaded, and a password provided, all documents to be sent to UCL CCTU should be encrypted, this can be done as follows:

- Using windows explorer select the documents that require encryption, for one patient only, right click and select the 7-Zip programme, then select 'Add to archive' (figure 1)
- The 'Add to archive' window will appear. Add a file name (patient study identifier) under 'Archive'(figure 2)
- In the encryption section, enter the password provided by UCL CCTU
- Tick 'Show Password' if required
- Please type the password. Do not copy and paste the password from other applications.
- Ensure that the encryption method is **AES-256** and press ok.

The documents will now only be accessible with the provided password.

Only encrypted copies stored/sent ensure that only authorised personnel will be able to access the information. Once the documents have been encrypted, consider deleting all un-encrypted copies.

EVERREST

UCL Comprehensive Clinical Trials Unit

UCL Clinical Trials Unit

Document Title:	**Standard Operating Procedure for performing ultrasound examinations**
Document Type:	SOP
Document Number:	EVE002
Version Number:	Version 3.0
Effective Date (training can commence):	
Date of Final Release:	
Review Date:	1 year from initial release and 2 years thereafter
(If applicable) Version of SOP being superseded:	2.0

Author	Title	Signature	Date
Dr. Anna David	Principal Investigator, Reader and Consultant in Obstetrics and Maternal Fetal Medicine		17 March 2015.
Reviewed By		Signature	Date
Professor Donald Peebles	UCLH Co-Investigator, Professor and Head of the Research Department of Maternal Fetal Medicine		
Professor Karel Marsal	ULUND Principal Investigator, Professor at the Department of Obstetrics and Gynaecology		19 March 2015
Professor Kurt Hecher	UKE Principal Investigator, Professor and Chairman of the Department of Obstetrics and Fetal Medicine		
Dr. Eduard	IDIBAPS Principal Investigator,		

UCL Clinical Trials Unit

Document Title:	**Standard Operating Procedure for performing ultrasound examinations**
Document Type:	SOP
Document Number:	EVE002
Version Number:	Version 3.0
Effective Date (training can commence):	
Date of Final Release:	
Review Date:	1 year from initial release and 2 years thereafter
(If applicable) Version of SOP being superseded:	2.0

Author	Title	Signature	Date
Dr. Anna David	Principal Investigator, Reader and Consultant in Obstetrics and Maternal Fetal Medicine	[signature]	17 March 2015
**Reviewed by**		**Signature**	**Date**
Professor Donald Peebles	UCLH Co-Investigator, Professor and Head of the Research Department of Maternal Fetal Medicine		
Professor Karel Marsal	ULUND Principal Investigator, Professor at the Department of Obstetrics and Gynaecology		
Professor Kurt Hecher	UKE Principal Investigator, Professor and Chairman of the Department of Obstetrics and Fetal Medicine	[signature]	19/03/15
Dr. Eduard	IDIBAPS Principal Investigator,		

Gratacós	Head and Professor of the Department and Research Centre in Maternal Fetal Medicine		
Dr Francesc Figueras	Principal investigator for IDIBAPS Department and Research Centre in Maternal Fetal Medicine		
Anna Morka	Trial Manager	Anna Morka	10 Mar 2015
Julie Bakobaki	Clinical Operations Manager	[illegible]	10 MARCH 2015
Approvals	Title	Signature	Date
Dr. Anna David	Chief Investigator	[illegible]	17 march 2015.
